# Detection of Greenhouse Gas Precursors from Diesel Engines Using Electrochemical and Photoacoustic Sensors

**DOI:** 10.3390/s101109726

**Published:** 2010-11-01

**Authors:** Geórgia Mothé, Maria Castro, Marcelo Sthel, Guilherme Lima, Laisa Brasil, Layse Campos, Aline Rocha, Helion Vargas

**Affiliations:** Laboratório de Ciências Físicas LCFIS–Universidade Estadual do Norte Fluminense UENF, Av. Alberto Lamego 2000, Campos dos Goytacazes, Rio de Janeiro, Brasil; E-Mails: georgiamothe@gmail.com (G.M.); sthel@uenf.br (M.S.); guiuenf@yahoo.com.br (G.L.); laisa.riguetibrasil@gmail.com (L.B.); laysecampos@hotmail.com (L.C.); amraline@hotmail.com (A.R.) Vargas@uenf.br (H.V.)

**Keywords:** diesel engines, electrochemical sensors, photoacoustic sensors

## Abstract

Atmospheric pollution is one of the worst threats to modern society. The consequences derived from different forms of atmospheric pollution vary from the local to the global scale, with deep impacts on climate, environment and human health. Several gaseous pollutants, even when present in trace concentrations, play a fundamental role in important processes that occur in atmosphere. Phenomena such as global warming, photochemical smog formation, acid rain and the depletion of the stratospheric ozone layer are strongly related to the increased concentration of certain gaseous species in the atmosphere. The transport sector significantly produces atmospheric pollution, mainly when diesel oil is used as fuel. Therefore, new methodologies based on selective and sensitive gas detection schemes must be developed in order to detect and monitor pollutant gases from this source. In this work, CO_2_ Laser Photoacoustic Spectroscopy was used to evaluate ethylene emissions and electrochemical analyzers were used to evaluate the emissions of CO, NO_x_ and SO_2_ from the exhaust of diesel powered vehicles (rural diesel with 5% of biodiesel, in this paper called only diesel) at different engine rotation speeds. Concentrations in the range 6 to 45 ppmV for ethylene, 109 to 1,231 ppmV for carbon monoxide, 75 to 868 ppmV for nitrogen oxides and 3 to 354 ppmV for sulfur dioxide were obtained. The results indicate that the detection techniques used were sufficiently selective and sensitive to detect the gaseous species mentioned above in the ppmV range.

## 1. Introduction

Air pollution is currently a major concern of society, with serious economic impacts and causing serious damage to human health. The development of new methods of pollutant detection has acquired increasing relevance. Pollution emitted by industry, vehicles, power plants and other sources has stimulated the development of effective methods of air pollution control. These methods have to be very selective and sensitive in order to successfully solve the ecological problems. The atmospheric pollution by numerous trace gases is extremely important, as it can cause serious consequences to human health and the environment, causing problems as acid rain, photochemical smog and global climatic changes, which are produced principally by the intensification of the so-called greenhouse effect [1–15]. The knowledge of temporal and spatial distribution of air pollutants is a prerequisite for the understanding of the complex chemical processes occurring in the atmosphere and thus for the verification of the theoretical models or for the evaluation of pollution reduction strategies. A better comprehension of those phenomena and the validation of the air pollution prediction models requires knowledge of the involved chemical species and their spatial and temporal distributions. Therefore, the use of suitable analytical techniques is necessary for the identification of the atmospheric components and to determinate their trace concentrations. A trace sensor of atmospheric pollutants must meet a set of fundamental requisites. High selectivity is necessary to distinguish the gas species present in a multicomponent gas mixture, such as air and high sensitivity is essential to detect very low concentrations of substances. A large dynamic range is important to monitor the gas components at high and low concentrations with the same instrument. In addition, a good time resolution ensures the possibility of on-line analyses controlled by a computer.

The diesel engine has great significance in the day-to-day life of modern society. It is largely used in our land and sea transport, and provides electrical power, being also used in many farming, construction and industrial activities. Diesel is an important part of the public and private transportation sector and its use can be expected to continue and increase in the near future. However, diesel is one of the largest contributors to environmental pollution problems worldwide and this problem can only get worse, as the number of diesel powered vehicles is increasing significantly. Diesel emissions contribute to the occurrence of many cardiovascular and respiratory diseases and cancer, besides causing air, water and soil pollution, visibility reductions and global climate changes [16–19].

In this context, the present work evaluated the emissions of diesel powered vehicles [20–21] in Campos dos Goytacazes, a city located in the northern Rio de Janeiro state, Brazil. This city has an area of 4,031.910 km^2^ this is ten times the State of California and a population of 426,154 inhabitants [22]. The analyzed vehicles are used in urban and cargo transport and produce a large amount of air pollutants, such as nitrogen oxide (NO_x_) and sulfur dioxide (SO_2_), generators of acid rain and harmful to human health [23–28], carbon monoxide (CO), another pollutant emitted by diesel engines, is not considered a direct greenhouse gas, but it is able to influence the production of methane and tropospheric ozone, which are important greenhouse gases [29–37]. Diesel engines also produce volatile organic compounds (VOCs), such as ethylene [43–45]. It is well known that ethylene is a reactive pollutant, since it is an unsaturated organic compound [46]. For this reason, this chemical species is a precursor for the generation of the tropospheric ozone [47–49], which is present in photochemical smog and directly affects human health. Tropospheric ozone can also trigger serious respiratory problems and cardiovascular effects [38–42]. Besides, it is a powerful greenhouse gas, whose formation is greatly potentiated by the incidence of sun radiation and the presence of nitrogen oxides (NO_x_) [50,51]. According to the Intergovernmental Panel of Climatic Changes (IPCC), ozone has a positive radiative forcing of about 0.35 W/m^2^, being, therefore, an important source of global warming. Other species, such as dinitrogen monoxide (N_2_O) and methane (CH_4_) have positive radiative forcing of about 0.16 W/m^2^ and 0.48 W/m^2^, respectively [2]. Tropospheric ozone is present in the largest cities in all world and its generation is associated with the emissions of VOCs by urban and cargo transports, with a significant contribution of ethylene. The methodologies based on photothermal techniques, mainly CO_2_ laser photoacoustic spectroscopy, have suitable characteristics to detect trace gases (ethylene). Photoacoustic spectroscopy is widely used for the detection of several gases in the concentration range of ppbV and sub-ppbV [52–61]. This methodology is based on the generation and detection of pressure waves (sound) inside a resonant cell, where the gas samples are placed. These samples are exposed to the incidence of modulated radiation, absorbing it at determined wavelengths. The resonant absorption of radiation generates a modulated heating in the sample and, therefore, a sound signal is produced and detected by highly sensitive microphones, inside the cell. These microphones convert the sound signal into an electric signal, which is filtered and detected by a lock-in amplifier. In this work, a detection limit in the ppbV range for ethylene was achieved and laser photoacoustic spectroscopy was used to detect and monitor ethylene concentrations emitted by diesel powered engines in the ppmV range. Sensors based on electrochemical principles were used to detect nitric oxide gas. It was also possible to detect CO, NO_x_ and SO_2_ emissions by diesel powered engines in the ppmV range.

## 2. Methodology

**Figure 1.**
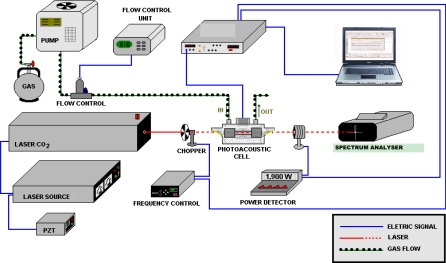
Scheme of the photoacoustic experimental setup.

The gas samples were collected from the exhaust of diesel powered vehicles and were stored in previously evacuated metallic canisters (SUMMA Andersen Instruments). The canisters are made of stainless steel and the samples were taken to the laboratory and coupled to our photoacoustic cell inlet. The gas sample was then pulled into this cell by a mechanical pump (Ambient Volatile Canister Sample AVOCS Graseby). Filters were used to remove the particulate matter larger than 2 μm. This collection was performed in two sequences in each vehicle: the first one with the bus engine turned on and without acceleration (*i.e.*, 1,000 rpm of rotation speed) and the second one, with the bus engine turned on and with acceleration (*i.e.*, 3,000 rpm of rotation speed). The gas samples were analyzed by a photoacoustic method at a pressure of 1 atm. Figure 1 shows the experimental setup. In conventional absorption spectroscopy, the absorption of the radiation power transmitted through the sample is measured. On the contrary, in photoacoustic spectroscopy, the absorbed power is determined directly via its heat and hence the sound produced in the sample. Actually, several laser-based methods such as photoacoustic and cavity-ring-down spectroscopy have been reported because they are much more sensitive [62,63]. Photoacoustic spectroscopic methods offer important advantages in pollutant gas monitoring. This technique is based on pressure changes in the sample, which is induced by rovibrational excitation of molecules and, subsequent, relaxation by collisions (heat). The pressure change is detected by one or more microphones placed inside a resonator pipe of a resonant photoacoustic cell through which, the air sample containing the molecules under consideration was flown. An acoustic signal is produced at the resonance frequency of about 2,400 Hz of our resonant cell, by a chopper modulation of the excitation laser beam. The resonance frequency value corresponds to the first longitudinal vibration mode. Our photoacoustic resonator is 67 mm long and has a diameter of 18 mm.

**Figure 2.**
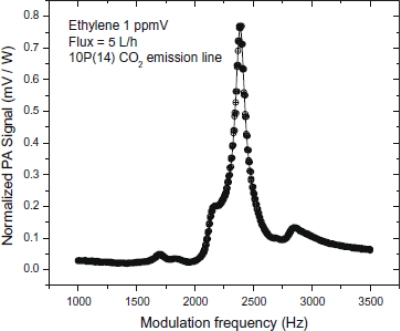
Resonance curve showing the photoacoustic signal variation with the chopper modulation frequency.

This resonance frequency value, as well as the Q-factor (Q = 24.7) of the resonant photoacoustic cell were obtained performing a photoacoustic signal sweep with the modulation chopper frequency between 1.0 kHz and 3.5 kHz (Figure 2).

This measurement was carried out using a certified gas mixture of 1.1 ppmV ethylene in N_2_ flowing into the cell at a rate of 5 L/h. The ethylene and nitrogen gases were calibrated and purchased from White Martins. The acoustic signal is detected by a microphone that generates an electric signal. This electric signal is pre-amplified and then detected by a lock-in amplifier (Stanford SR850) with a time constant of 300 ms. The lock-in response is registered in a microcomputer. A continuous wave CO_2_ infrared laser (Lasertech Group Inc., – LTG, model LTG150 626G), tuneable over about 80 different lines between 9.2 and 10.6 μm, with a power of 1.9W at the emission line 10P(14) (10.53 μm), with internal PZT (Piezoelectric Transducer), was employed as the excitation source. At this power level, no saturation effects of the photoacoustic signal were observed. These lines can be swept by a step motor controlled by a microcomputer. Within this spectral region, many small molecules show a unique fingerprint. The photoacoustic instrument used in this work has been developed for the detection of small concentrations of gases. All the measurements and the sample collection were made at room temperature. The measurements in this work were performed with multicomponent gas samples, as one would expect of samples collected from the ambient air, vehicle exhausts, *etc.* Therefore, the analysis of these samples was made for a number of *n* different species, rather than just one. This was accomplished by measuring the photoacoustic (PA) signal (S) at a set of wavelengths λ_i_ (i = 1, 2… m) chosen on the basis of the absorption spectra of the individual components to be detected. These individual absorption spectra were obtained from the HITRAN-PC database, [64] which calculates the absorption cross sections (σ) of a given molecule at different wave numbers k_i_ = 1/λ_i_ in a given interval. Thus, the expression used to determine the concentrations of a given component in the multicomponent gas mixture is:

(1) $$S_{\lambda i}=S_{i}={\mathrm{CP}}_{i}N_{\mathrm{tot}}\sum_{j=1}^{n}c_{j}\sigma_{\mathrm{ij}}$$

with i = 1, 2, ........., m; j = 1, 2, ......., n and m > n. Here, P_i_ = P(λ_i_) represents the laser power at wavelength λ_i_ and c_j_ is the concentration of the gas component *j* with absorption cross section σ_ij_ at λ_i_. N_tot_ is the total number density of molecules in the mixture and was considered to be typically ∼10^19^ cm^−3^ [65]. The absorption cross section σ_ij_ is related to the photoacoustic generation efficiency of each gas component for each CO_2_ laser line. The sum is taken over the *n* components present in the sample. The constant C is the so-called cell constant and it depends, as well as the detection sensitivity, on the cell geometry, the microphone response and on the nature of the acoustic mode [65,66].

### 2.1. Photoacoustic Cell Calibration and Sensitivity Measurements

**Figure 3.**
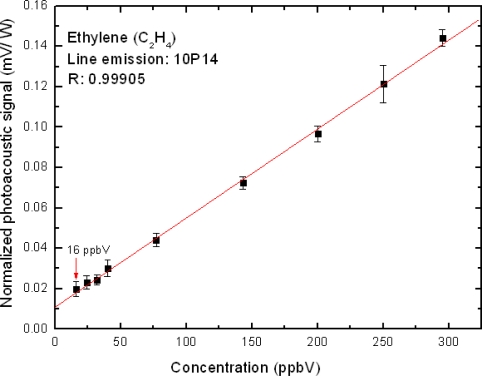
Calibration curve for ethylene (detection limit).

The calibration and sensitivity measurements of our photoacoustic cell were performed by obtaining the cell coupling constant C in the Equation (1). This was performed by taking a 1.1 ppmV certified mixture of ethylene in N_2_ and diluting it in nitrogen until the least concentration achieved (about 16 ppbV), as shown in Figure 3. Thus, a calibration curve relating the normalized PA signal (SN = S/P) and the ethylene concentration could also be obtained. As this function has a linear behavior is possible extend this linearity to ppmV levels. This calibration line follows the expression:

(2) $$S_{i}={\mathrm{CP}}_{i}N_{\mathrm{tot}}c_{\mathrm{ref}}\sigma_{i}$$

The absorption cross section σ of ethylene is well known at the 10P(14) (949.51cm^−1^) CO_2_ laser line (σ = 170 × 10^−20^ cm^2^). Hence, the C constant value was then obtained from the eq. (2), which yielded 40.2 V.cm/W. The unity of the cell coupling constant was furnished by the manufacturer of our photoacoustic cell (Prof. Markus W. Sigrist).

#### 2.2. Electrochemical Sensors

Electrochemical sensors are important tools for the detection of gaseous species due to their portability, easy automation and low cost. They also enable performing measurements *in situ* and in real time. The electrochemical sensors (Figure 4) wethis work are composed of a sensing electrode, a counter electrode, a reference electrode and a reagent electrolyte inserted between the electrodes. Furthermore, a barrier permeable to gas, also known as hydrophobic membrane, must cover the sensor to avoid the entry of undesirable gases and water and to control the amount of gaseous molecules that reach the electrode surface. The choice of material of the electrode depends on the type of gas to be detected and usually noble metals like gold and platinum are used. The function of the electrolyte is to facilitate the reaction and the effective ionic charge transport through the electrodes, beside providing an element of selectivity because it does not interact with other molecules.

When the gas enters the sensor, it will react with the electrodes in an oxidation-reduction process. As the electrodes are connected to a resistor, an electric current is generated between the cathode and the anode. The current generated is proportional to the concentration of gas [67,68].

A Tempest 100 (Telegan Gas Monitoring TP20729) analyzer was used in this work, composed of electrochemical sensors (Citycell Gold Class) capable of measuring simultaneously through of the manifold system the concentration of NO_x_ (0–1,000 ppmV), CO (0–10,000 ppmV) e SO_2_ (0–2,000 ppmV). The calibration of the Tempest 100 unit is performed annually by the Confor Instrumentos company (São Paulo-SP, Brazil) using gas calibration (Gama Gases or Air Products) obtaining a uncertainly of ±5 ppmV in 03/11/2009. The probe of the Tempest 100 is 2.5 meters in length and is capable of withstanding a temperature up to 700 °C, although the temperature of the sample may damage the sensor. The measurements were performed directly in the exhaust tube of these vehicles due to the portability of this equipment.

## 3. Results and Discussion

The photoacoustic (PA) measurements performed in this work (figures 2 and 3) clearly show that our resonant PA cell has an excellent performance, with low electronic noise level (signal/noise ∼44 for minimum concentration measurements of ethylene in exhaust) and a great stability in its resonant frequency (around 2,400 Hz), as well as a considerable linearity of the PA signal when diluting the ethylene in nitrogen. The CO_2_ laser emission line 10P(14), which is the strongest ethylene absorption line, was used for these measurements and no interference with the ethylene signal from peaks belonging to other species, such as water vapor and CO_2_ was detected. Due to this fact chemical filters such as calcium chloride (CaCl_2_) to filter water vapor and potassium hydroxide (KOH) to filter carbon dioxide were used in our experiments. Moreover the absorption coefficients for these chemical species in the CO_2_ laser wavelength range are very small. With those calibrations, it was possible to detect ethylene in the 16 ppbV range.

As this function has a linear behavior is possible to extend this linearity to ppmV levels. Therefore, PA spectroscopy revealed itself as a very precise and sensitive technique, since it was able to detect and monitor ethylene in our samples. Table 1 lists all vehicles from where the gas samples were collected for ethylene detection. In group A (buses), six buses were chosen to illustrate the feasibility of the photoacoustic technique to detect ethylene concentrations, obtained in the range from 3 ppmV to 45 ppmV for high and low operation regimes (Figure 4). The important feature demonstrated in this figure is that, as it would be expected, the older the bus the larger is its ethylene concentration emission, mainly in the higher rotation speed of the engine (1,000–3,000 rpm). Buses 2 and 5, the newest ones, emitted less ethylene than the other buses.

**Figure 5.**
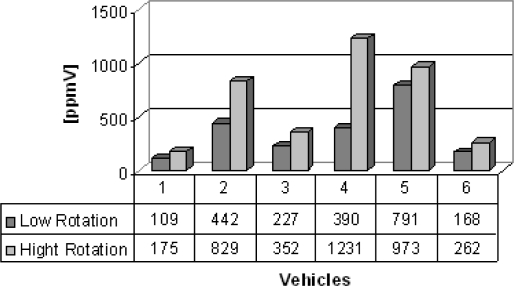
Carbon monoxide gas concentration [ppmV].

**Figure 6.**
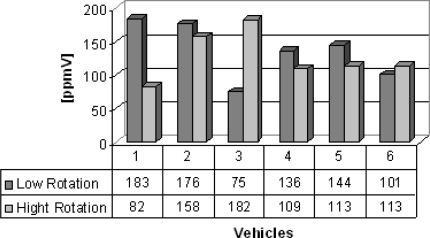
Nitrogen oxide gas concentration [ppmV].

**Figure 7.**
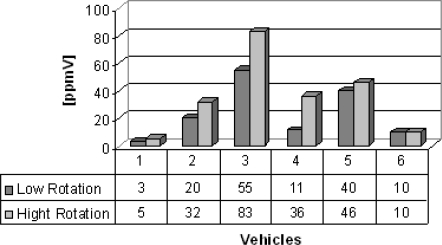
Sulfur dioxide gas concentration [ppmV].

Group B are vans from which the gas samples were collected. Six vans were chosen to illustrate the feasibility of the electrochemical sensors, which succesfully detected carbon monoxide, nitrogen oxide and sulfur dioxide detection and the results are shown in figures 5–7. Carbon monoxide concentrations from 109 to 1,231 ppmV, nitrogen oxides concentrations from 75 to 183 ppmV and sulfur dioxide concentrations from 3 to 83 ppmV were detected in the exhaust in the both engine operation modes. Figure 6, concerning the issue of nitrogen oxides NOx, shows that this species behaves anomalously as it is coming from the interaction of nitrogen and oxygen from the air during the burning of fuel in the combustion chamber, thus depending on the temperature of combustion. Thus, the operation mode of the engine (high or low speed) becomes an important factor in emissions of NOx. Possibly these results were generated by the difficulty of controlling an experiment to be performed in real time and *in situ*, because it represents the dynamics of the transport of a city, because some collections were made in already overheated engines and others in cold engines.

**Figure 8.**
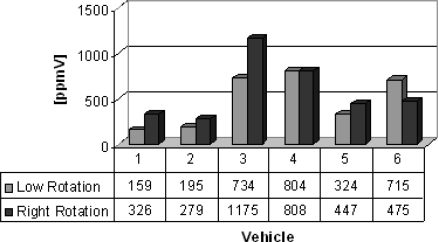
Carbon monoxide concentrations [ppmV].

**Figure 9.**
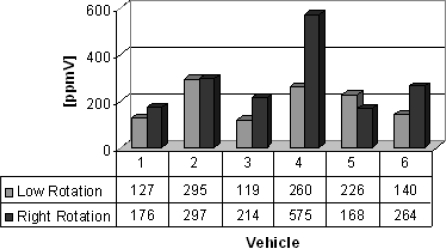
Nitrogen oxides concentrations [ppmV].

Group C lists vehicles from which the gas samples were collected. Six different vehicles (buses and trucks) were chosen to illustrate the feasibility of the electrochemical sensors. The results are shown in figures 8 and 9. Carbon monoxide concentrations from 159 to 1,175 ppmV and nitrogen oxides concentrations from 119 to 575 ppmV were obtained in both engine operation modes.

**Figure 10.**
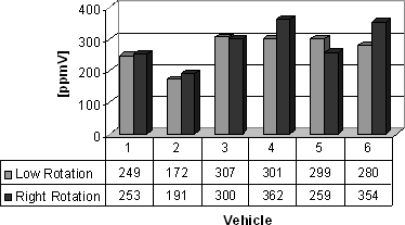
Sulfur dioxide gas concentrations [ppmV].

**Figure 11.**
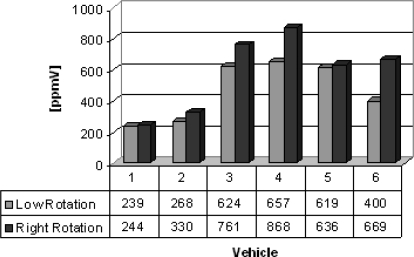
Nitrogen oxides gas concentrations [ppmV].

Group D lists vehicles (buses) from which gas samples were collected. Six buses (two new and four very old ones) were evaluated to know the sulfur dioxide and nitrogen oxides emitted. The results are shown in figures 10 and 11. Sulfur dioxide concentrations from 192 to 362 ppmV and nitrogen oxides concentrations from 239 to 868 ppmV were obtained. The older buses emit more sulfur dioxide and nitrogen oxide in the both engine operation modes.

In fact, the analysis of diesel powered vehicles indicates that many factors can contribute to the emission of pollutant gases from diesel powered engines. These factors include the year of manufacture, brands and models, engine power, fuel quality and engine regulation and maintenance, cleaning of fuel nozzles and replacement of air filters (subjective factors).

Given the numerous variables involved in the emmision process in vehicle engines, it becomes difficult to find a correlation between the concentrations of emissions and the various chemical species involved in this process. This work was aimed at using the photoacoustic spectroscopy and electrochemical sensors to detect polluting gases, and both techniques proved sensitive and selective. Photoacoustic spectroscopy has a wide dynamic range of detection (ppbV to ppmV), as its theoretical model predicts, the linearity between photoacoustic signal and concentration of gas. The eletrochemical sensors are grouped in a single device, but are independent of each other, that is by its selective elements (hidrophobic membrane and electrolyte) ensuring selectivity in the detection of various gases. They have a range of about ppmV, with an uncertainty of 5 ppmV, and as most of the results obtained in this work are the order of tens to hundreds of ppmV, this technique is sensitive within the range of concentrations obtained in this experiment.

In this work there were, in a general, great amounts of ethylene (C_2_H_4_), carbon monoxide (CO), nitrogen oxides (NO_x_) and sulfur dioxide (SO_2_) emissions in the ppmV range for all the vehicles investigated.

This research results indicate the necessity of a transport renewal program in Brazil, because it is a large country, with a great number of diesel powered buses, (hundreds of thousands) and diesel powered trucks (one million) that are on average 20 years old. The increasing number of vehicles makes the atmospheric pollutant emissions worse. In addition, mass transport in the country is predominantly by road. This means that a possible increase in pollutant gases emissions to the atmosphere is very likely. Since Brazil is a tropical country with strong sun irradiation all year long, ethylene emissions by buses and trucks will contribute to potentiate tropospheric ozone (greenhouse gase) formation in Brazilian cities. Another problem is the lack of a specific legislation for some pollutant gases, since the present legislation is only concerned with the total amount of emitted hydrocarbons and it does not discriminate between some important pollutants, such as ethylene. However, diesel powered vehicles emit many other pollutant gases, such as CO, NO_x_ and SO_2_, which generate acid rain, besides being harmful to human health. This complex reality is present in many countries, but it is worse in emerging countries, where great part of the cargo and public transportation vehicles use diesel engines. Therefore, there is a general concern with the atmospheric pollution in the country at the government and organized civilian society levels.

## 4. Conclusions

In conclusion, in this work photoacoustic measurements of ethylene and electrochemical sensor measurements of carbon monoxide, nitrogen oxides and sulfur dioxide emitted by diesel vehicle engines were presented. These sensors provided a sufficiently selective detection of the gases mentioned, they are individualized and offer sensitivity in the ppmV range. This leads to the problem that the total amount of ethylene emitted to the atmosphere is not taken into account. Our experimental results clearly show that older buses produce more of the pollutant gas ethylene in relation to the newer ones and, at the same time, make the efficiency of the photoacoustic spectroscopy in detecting and monitoring gases evident. This technique revealed itself as an extremely sensitive and selective one as it was able to detect ethylene in the ppbV range. This way, this physical technique has shown itself very suitable to study the collected gas samples. As mentioned above, Brazil is a huge country in area and population which makes the problem of the spatial distribution of the atmospheric pollution in the country worse, either in the smaller cities or in the bigger urban centers. Possibly, great part of the troposphere ozone generation arises from the large number of buses and trucks circulating in Brazilian cities. Therefore, there has been a great effort to turn the Brazilian transport system into a more sustainable one, through the adoption of governmental programs that stimulate the use of biofuels, such as alcohol and biodiesel, in substitution of fossil fuels, mainly diesel oil and gasoline.

Diesel oil is widely used as fuel around the world, especially in urban and cargo transportation. Thus, ozone precursors such as ethylene and nitrogen oxides and carbon monoxide occur virtually in all countries and, consequently, ozone, an important greenhouse gas, creates a great environmental problem. Of course, this problem is amplified in countries with high incidence of sun radiation, such as for example Brazil and African countries.

## Figures and Tables

**Figure 4.**
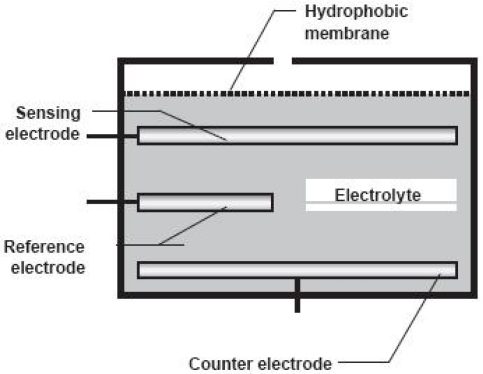
Detection scheme of an electrochemical sensor.

**Figure 4.**
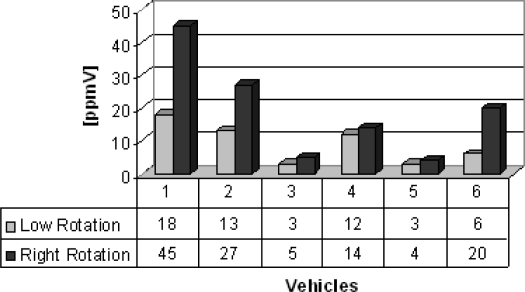
Ethylene gas concentration [ppmV].

**Table 1.** Lists all vehicles from where the gas samples were collected, divided into groups.

**Group**	**Number**	**Vehicle**	**Year**	**Group**	**Number**	**Vehicle**	**Year**
**A**	1	Bus	1996	**C**	1	Truck	2006
	2	Bus	1997		2	Truck	2006
	3	Bus	2006		3	Truck	2002
	4	Bus	2001		4	Bus	2004
	5	Bus	2006		5	Bus	2005
	6	Bus	2003		6	Bus	2004
**B**	1	Van	2004	**D**	1	Bus	2006
	2	Van	1997		2	Bus	2006
	3	Van	2007		3	Bus	1980
	4	Van	2001		4	Bus	1989
	5	Van	1999		5	Bus	1985
	6	Van	1997		6	Bus	1981
